# Interactions Among Multiple Quantitative Trait Loci Underlie Rhizome Development of Perennial Rice

**DOI:** 10.3389/fpls.2020.591157

**Published:** 2020-11-12

**Authors:** Zhiquan Fan, Kai Wang, Jianglei Rao, Zhongquan Cai, Li-Zhen Tao, Yourong Fan, Jiangyi Yang

**Affiliations:** ^1^ State Key Laboratory for Conservation and Utilization of Subtropical Agro-Bioresources, College of Life Science and Technology, Guangxi University, Nanning, China; ^2^ College of Agriculture, Guangxi University, Nanning, China; ^3^ State Key Laboratory for Conservation and Utilization of Subtropical Agro-Bioresources, South China Agricultural University, Guangzhou, China

**Keywords:** clonal plants, gene mapping, perennial crop, quantitative trait locus, selective genotyping mapping

## Abstract

Perennial crops have some advantages over annuals in soil erosion prevention, lower labor and water requirements, carbon sequestration, and maintenance of thriving soil ecosystems. Rhizome, a kind of root-like underground stem, is a critical component of perenniality, which allows many grass species to survive through harsh environment. Identification of rhizome-regulating genes will contribute to the development of perennial crops. There have been no reports on the cloning of such genes until now, which bring urgency for identification of genes controlling rhizomatousness. Using rhizomatous *Oryza longistaminata* and rhizome-free cultivated rice as male and female parents, respectively, genetic populations were developed to identify genes regulating rhizome. Both entire population genotyping and selective genotyping mapping methods were adopted to detect rhizome-regulating quantitative trait loci (QTL) in 4 years. Results showed that multiple genes regulated development of rhizomes, with over 10 loci related to rhizome growth. At last, five major-effect loci were identified including *qRED1.2*, *qRED3.1*, *qRED3.3*, *qRED4.1*, and *qRED4.2*. It has been found that the individual plant with well-developed rhizomes carried at least three major-effect loci and a certain number of minor-effect loci. Both major-effect and minor-effect loci worked together to control rhizome growth, while no one could work alone. These results will provide new understanding of genetic regulation on rhizome growth and reference to the subsequent gene isolation in rice. And the related research methods and results in this study will contribute to the research on rhizome of other species.

## Introduction

Global demand for food is increasing, accompanying decrease of arable land (Niihama et al., 2015). Crop yield became the focus of our attention, neglecting social and environmental consequences of these food production systems. In order to increase the yield, more and more fertilizers, pesticides and water are consumed, especially in developing countries. Most of the conventional crops are annual plants. Annual crops require more water, fertilizer and labor costs, and are prone to disrupt biodiversity and ecosystem function than more rustic perennial crops (Batello et al., 2014). Perennial crops, compared with annual counterparts, tend to have longer growing seasons and deeper rooting depths, which was helpful to intercept and utilize more precipitation (Randall et al., 1997; Wallace, 2000; Dohleman and Long, 2009; Glover et al., 2010). In the other side, perennial crops can prevent erosion, save water, and nutrients, all of which are benefit for the maintenance of healthy soil ecosystems over the long-term and confer ecological benefits (Cox, 2014).

Perenniality in grass species originates mainly from vegetative organs with property of indeterminate growth, such as rhizome or stolon. Rhizome, a kind of root-like underground stem which grows horizontally, is a key organ that distinguishes annual cultivar from their perennial wild relatives. As a critical component of perenniality, rhizome enables grasses survive through harsh conditions, such as drought or cold, and also renew quickly at the next growing season (Hu et al., 2003; Westerbergh and Doebley, 2004; He et al., 2014). Genes controlling rhizome proliferation can be used to breed perennial cereal crops, forage and turf grasses, and contribute to control the invasive perennial weeds. However, genetic mechanisms of rhizomatousness have still not been fully elucidated in any plants (Yun et al., 2014).

Rhizome confers not only perenniality but also clonality to plants. For rhizomatous plants, perenniality closely interconnected with clonality. Plants can be regarded as an assembly of many modules (de Kroon et al., 2005). If these modules can iterate themselves spontaneously and thus produce potentially independent vegetative propagule, these plants can be called clonal plants (Dong, 1996; de Kroon and van Groenendael, 1997; Hutchings and Wijesinghe, 1997). Compared with ordinary plants, clonal plants have many unique properties, such as longevity, mobility, dual reproduction (i.e., both sexual and asexual reproduction), and clonal physiological integration (Dong and de Kroon, 1994; Dong and Pierdominici, 1995; Hutchings and Wijesinghe, 2008). A genetic individual (also called clone) is comprised of many ramets (vegetative daughter plants), which can spread out and exchange resources through spacers (physiological integration), and these spacers can be rhizomes, stolons or roots (de Kroon and van Groenendael, 1997; Yang et al., 2012). Among these spacers, rhizome is of great significance for many valuable grass species to survive and expand, which greatly improves competitiveness of the clone in the natural environment (Paterson et al., 1995). Stolon is a kind of specialized stem, which grows at the soil surface and forms adventitious roots at the nodes. For the exposure to sun and dry air during the dry season, stolon has less drought tolerance than underground rhizome (Sacks, 2014). For the same reason, it is also probably less tolerant of cold.

As a kind of clonal plant, perennial rice can quickly take advantage of environmental resources *via* horizontal rhizome extension. And they need less water and labor than annual rice and can protect soil from erosion (Sacks, 2014). Moreover, perenniality have the potential to fix heterosis, besides apomixes, but perennial hybrid rice would be easier to achieve than apomictic ones (Hu et al., 2003). Rhizome and stolon can both be utilized to breed perennial rice, but rhizome is superior to stolon for the reasons mentioned above. Therefore, rhizome is the most logical pattern of perennial rice breeding (Tao and Sripichitt, 2000), which brings urgency for identification of genes controlling rhizomatousness.

There are four kinds of perennial wild rice among the genus *Oryza*: *O. longistaminata*, *O. officinalis*, *O. australiensis*, and *O. rhizomatis*. *Oryza longistaminata*, distributed widely over Africa, is characterized by long anthers and strong rhizomes (Ghesquiere, 1985). It is the unique rhizomatous wild rice that has the same AA genome as cultivated rice, *O. sativa* (Hu et al., 2003), which will facilitate introgression of rhizomatousness from wild rice to cultivar.

It was initially suggested that rice rhizome development was controlled by a single locus *Rhz*, which was closely linked to the *liguleless* locus on chromosome 4 (Maekawa et al., 1998). Using an F_2_ population derived from the cross between *O. sativa* cultivar RD23 (an *indica* rice from Thailand) and an *O. longistaminata*, two dominant-complementary loci (*Rhz2* and *Rhz3*) were identified to largely control rhizomatousness (Hu et al., 2003). A single recessive mutation at either *Rhz3* or *Rhz2* would shut off rhizome expression (Hu et al., 2003). But follow-up studies indicated that there were still 5–8% plants had rhizomes, although they had RD23 (with recessive allele) homozygous genotypes at both *Rhz2* and *Rhz3* (Hu, 2010). Moreover, some plants had no rhizomes even when their *Rhz2* and *Rhz3* alleles were both *O. longistaminata* homozygous (Hu, 2015, presentation entitled “Progress in Perennial Rice Breeding and Genetics” accessible from http://pwheat.anr.msu.edu/2015/02/). These results demonstrated that only one or two quantitative trait loci (QTL) should not be enough to expound the complexity of rhizome regulation. And we hypothesized that there are some more genes regulating the rhizome development of rice. In order to find these genes, we chose *japonica* rice (Balilla) to hybridize with *O. longistaminata*. *Japonica* cultivars are easier to achieve genetic transformation than *indica* cultivars (Lin and Zhang, 2005). Our results showed that over 10 loci involved, and both major-effect and minor-effect loci worked together to control rhizome growth.

## Materials and Methods

### Plant Material and Field Experiments

With the African wild rice *O. longistaminata* (WYD-108) as male and *O. sativa* cultivar Balilla as female parents, respectively, mapping populations were prepared. Rhizome-free Balilla is a *japonica* rice (temperate rice with cool tolerance) introduced from Italy in 1958. WYD-108, from the National Germplasm Resources Nursery (wild rice) of China, is a rhizomatous wild rice imported from western Africa. Hybrid F_1_ between these two parents demonstrated the best rhizome growth among all cross combinations in our study (Fan et al., 2020).

**Figure 1:**
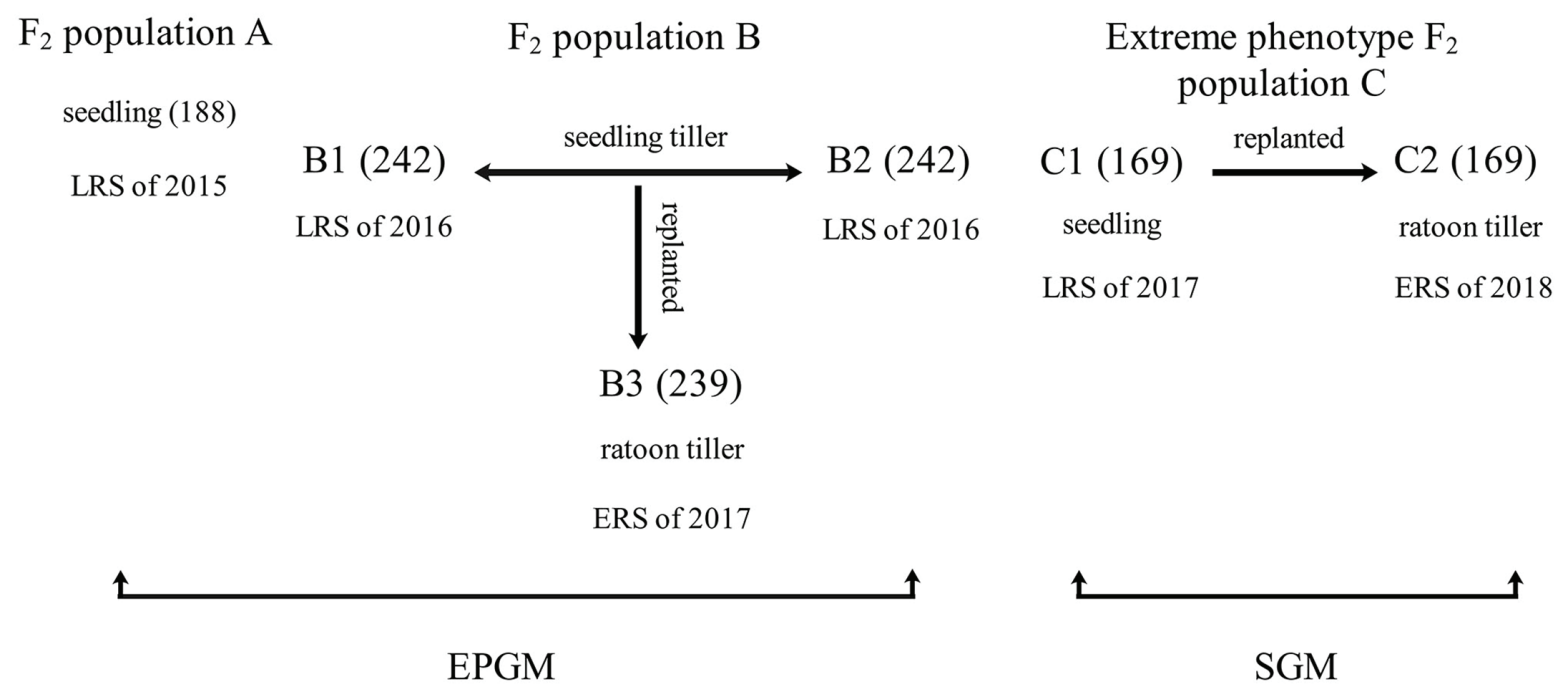
Details of the mapping population. Quantitative trait loci (QTL) mapping was performed using six phenotypic data of three populations from 2015 to 2018 with two different methods. Plants from two of these groups, A and B, had a continuous phenotype and were mapped by entire population genotyping mapping (EPGM) method. Population C comprised a total of 169 individuals with the longest and the shortest rhizome extension distance (RED), selected from 5,340 F_2_ rice plants with continuous phenotype. The phenotype of population C is discontinuous and selective genotyping mapping (SGM) method was used. Population A with 188 plants was planted in late rice season (LRS) of 2015. Population B was separated into two biological repeats (B1 and B2) *via* vegetative propagation of seedling tillers in the LRS of 2016 and had 242 individual plants; B3 was replanted from B1 in the early rice season (ERS) of 2017 *via* vegetative propagation of ratoon tillers. Population C was planted in the LRS of 2017 (C1) and ERS of 2018 (C2). C2 was replanted from C1 by vegetative propagation of ratoon tillers.

*Via* manual emasculation and hybridization followed by embryo rescue incubating on the aseptic culture medium (1/2 Murashige and Skoog Medium; Murashige and Skoog, 1962), the F_1_ plants of the Balilla/WYD-108 were obtained. And their seeds were harvested by bagged self-pollination individually. In order to identify QTL controlling rhizome phenotype, we developed three F_2_ populations (Figure 1). All F_2_ rice plants were grown in the field of Guangxi University, Nanning of China. The distance between each plant was 60 cm. Rice can be grown twice a year in Nanning: the early rice season (ERS) from March to July and the late rice season (LRS) from July to November. Three F_2_ populations were planted in different seasons and years (Figure 1). Population A was planted in the LRS of 2015 with total of 188 plants. Population B was separated into two biological repeats (B1 and B2, both have 242 plants) *via* vegetative propagation of seedling tillers in the LRS of 2016, and B3 (239 plants. A few plants failed to survive the winter in 2016–2017) was replanted from B1 in the ERS of 2017 *via* vegetative propagation of ratoon tillers. Population C containing 169 individual plants was planted in the LRS of 2017 (named C1) and ERS of 2018 (named C2). C2 was replanted from C1 by vegetative propagation of ratoon tiller.

### Phenotypic Evaluation

The average rhizome extension distance (RED) in soil surface was investigated in flowering (refer Hu et al., 2003) of most plants. Because the mother plant did not grow descendant ramets in all directions (probably due to alternating presence of axillary buds embedded in the alternate leaves), only the two farthest RED of each plant were measured and averaged.

### PCR Amplification and Molecular Marker Development

Total genome DNA was extracted from fresh leaf tissues using the CTAB method (Murray and Thompson, 1980). Compared with other codominant molecular makers, the PCR product bands of InDel (insertion/deletion) makers could be separated by agarose gel electrophoresis directly. We screened 653 InDel markers from Yang et al. (2015) and obtained 88 polymorphic InDel markers. In addition, eight polymorphic ones out of the 38 markers came from Niihama et al. (2015) and 66 polymorphic ones out of the 189 InDel markers provided by Luo (Zeng et al., 2016) were selected. Finally, a total of 162 InDel markers uniformly distributed on each chromosome were used for mapping. These InDel markers were named according to their chromosomal position. For example, C0429250 indicates that the marker located in 29,250 kb (kilo base pair) position of the fourth chromosome (C04). The chromosomal positions are based on the Os-Nipponbare-Reference-IRGSP-1.0 (IRGSP-1.0).^1^

### QTL Analysis

The genetic linkage maps of different F_2_ populations in 4 years were constructed by using R/qtl computer program (Broman, 2010). Windows QTL Cartographer 2.5 was used to calculate the effect value of each QTL.^2^ Two mapping methods including entire population genotyping mapping (EPGM) method and selective genotyping mapping (SGM) method were adopted to identify QTL affecting rhizome growth. EPGM (Edwards et al., 1987; Soller and Beckmann, 1990) method means that all planted F_2_ individuals were used to map QTL without phenotypic screens. SGM method (Lander and Botstein, 1989; Darvasi and Soller, 1992; Wingbermuehle et al., 2004; Sun et al., 2010) using the two-tailed or one-tailed extreme phenotype of the segregating population to analyze the linkage relationship between markers and QTL, which means that after phenotypic screening, some F_2_ plants instead of all individuals are used for recombination genetic map and identification of QTL. Populations A and B were mapped using the EPGM method, and all plants in the populations were used. Population C, unlike populations A and B, consisted of plants with two extreme phenotypes in RED. The extremely large phenotype has well-developed rhizome with RED more than 10 cm, while extremely small phenotype has poor-developed rhizome (RED closed to zero). And the 169 individual plants of population C with extreme RED selected from 5,340 F_2_ rice plants planted in the LRS of 2017. QTL of EPGM and SGM methods (Sun et al., 2010) were mapped by the Haley-Knott regression interval mapping method with setting the genotyping error rate at 0.01, QTL significance threshold of logarithm of odds (LOD) were declared at 5% based on 10,000 permutations (Broman and Sen, 2009).

## Results

### Phenotype of Parents and F_1_

**Figure 2:**
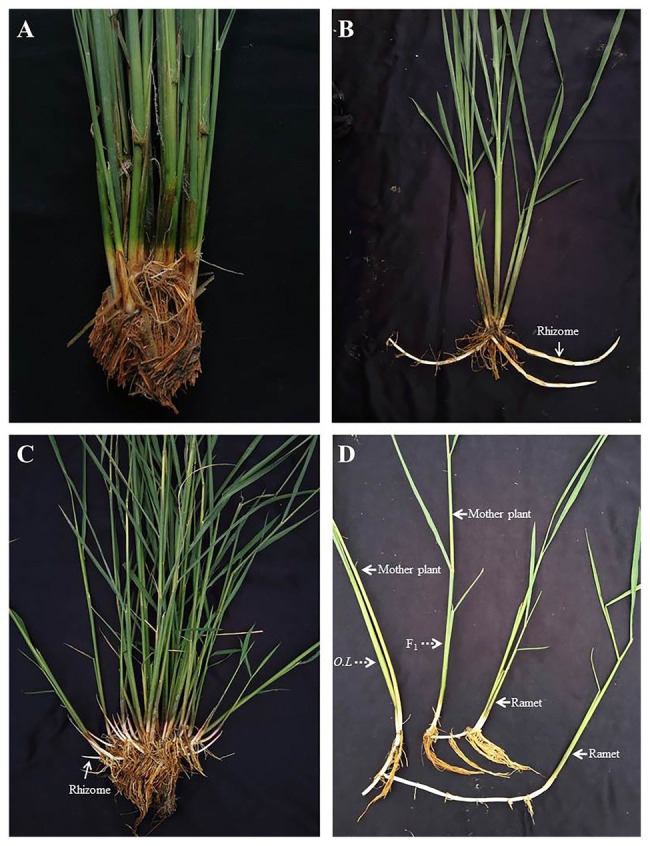
The phenotype of Balilla, *Oryza longistaminata* and F_1_ plants. **(A)** Female parent Balilla. **(B)** Male parent *O. longistaminata*. **(C)** Hybrid F_1_. **(D)** Difference of rhizome between F_1_ and *O. longistaminata*. F_1_, hybrid plants and *O.L*, *O. longistaminata*.

As a conventional cultivated rice, female parent Balilla is rhizome-free (Figure 2A) and the perennial wild rice *O. longistaminata* has flourishing rhizomes (Figure 2B). Their hybrid F_1_ had not only more rhizomes but also more tillers than *O. longistaminata* (Figure 2C), showing strong interspecific heterosis. But the rhizome length of hybrid F_1_ was shorter than that of *O. longistaminata*, resulting from fewer and shorter internodes of F_1_ rhizome (Figure 2D).

### Construction of Genetic Linkage Map

**Figure 3:**
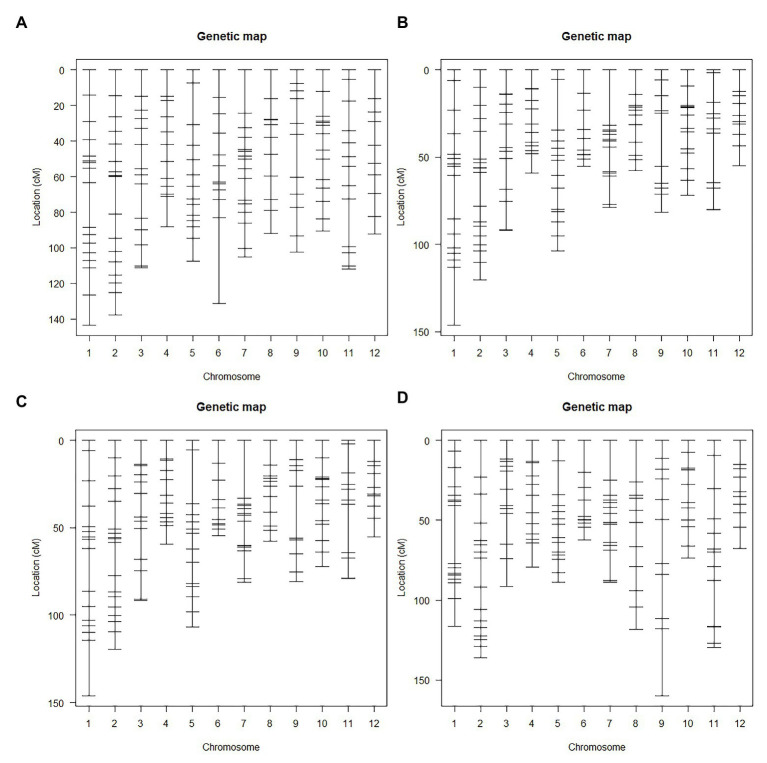
The genetic linkage map of F_2_ populations. **(A)** Genetic map of F_2_ population A. **(B)** Genetic map of F_2_ population B1 and B2. **(C)** Genetic map of F_2_ population B3. **(D)** Genetic map of F_2_ population C1 and C2.

Genetic linkage maps (Figure 3) constructed using InDel markers were based on different F_2_ populations (Figure 1). Genotypes of the F_2_ population were determined according to the gel bands of both the parents (as control) and F_1_ plant (to ensure codominance of markers). Markers were named according to their chromosomal position (refer section “Materials and Methods”). Genetic orders and locations of all the markers were nearly the same as the physical ones. Most markers with abnormal order and/or location were located in the centromere region. We also found 50 markers (30.9%), whose genotypic segregation distorted from the expected 1:2:1 Mendelian ratio. And the most significant segregation distortion was located on chromosome 7 and 12, implying the existence of potential loci for reproductive isolation (Yang et al., 2012).

### Identification of QTL for RED

**Figure 4:**
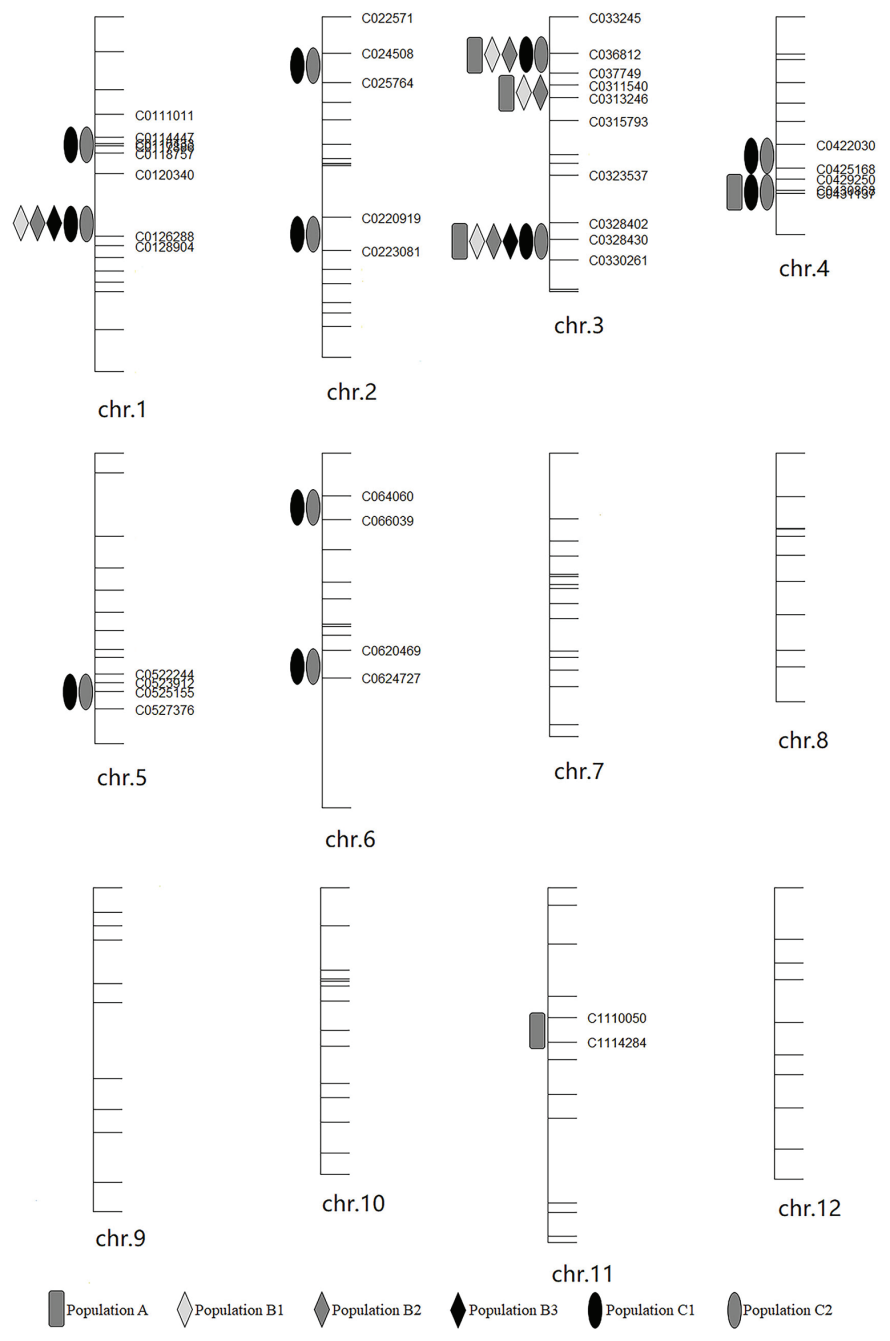
Positions of QTLs on the genetic linkage map in different year and season. The genetic map was constructed for population A.

**Figure 5:**
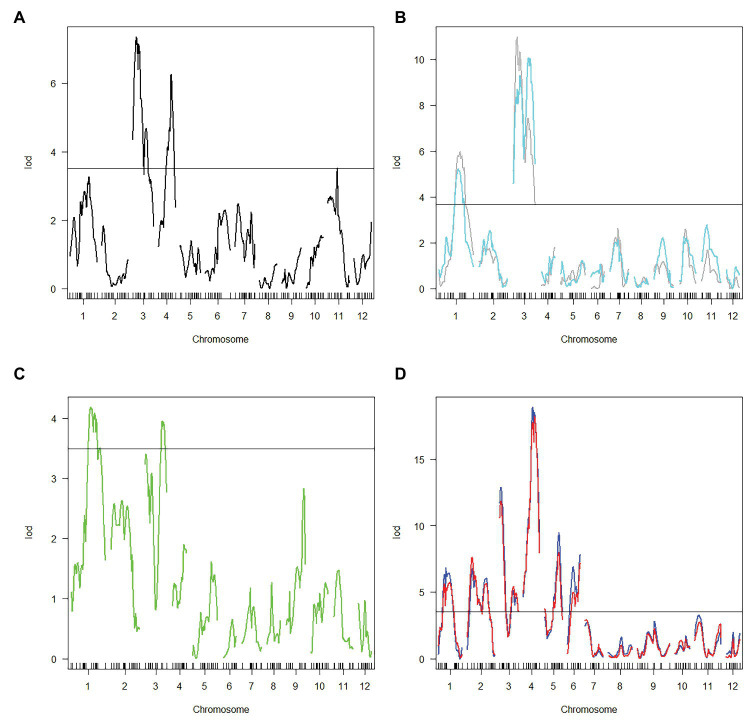
Logarithm of odds (LOD) score for QTL mapping in different populations. **(A)** Population A. **(B)** Population B1 and B2. Gray and cyan line represented population B1 and B2, respectively. **(C)** population B3. **(D)** Population C1 and C2. Blue and red line represented population C1 and C2, respectively. Horizontal line indicates the significant logarithm of odds threshold at 5% level of significance threshold based on 10,000 permutations.

Haley-Knott regression interval mapping method in R/qtl was used to identify the QTL for RED and a total of 13 QTL (Figure 4) were mapped with EPGM and SGM methods based on loci repeatability and LOD values above QTL significance threshold (5% based on 10,000 permutations) in three F_2_ populations. Naming of locus was referred to QTL nomenclature rules for rice (McCouch et al., 1997). The LOD calculated by Windows QTL Cartographer 2.5 was general accord with R/qtl (Supplementary Image 1, Supplementary Figures S1–S6), and the genetic effect of QTL is shown in Supplementary Table 1 (Supplementary Table S1). Six loci were identified in population A and B by EPGM method. Comparing QTL of F_2_ population A with B1 and B2, we found that only *qRED3.1*, *qRED3.2*, and *qRED3.3* could be detected repeatedly (Table 1). Population B1 and B2 planted at different plot in LRS (late rice season) of 2016 were vegetatively derived from the same F_2_ population (Figure 1), with similar QTL but different LOD score (Figure 5B). Compared with population B1 and B2, only two loci *qRED1.2* and *qRED3.3* were detected in B3 with much lower LOD (Figure 5C), indicating that rice planting season had a great impact on QTL mapping results, although B1, B2, and B3 were from the same F_2_ population (Figure 1). QTL results of EPGM were not always consistent in different years and seasons, with only a few loci could be detected repeatedly (Table 1). In view of this, individuals with extreme phenotypic differences were selected to map QTL hereafter.

**Table 1:** QTL affecting rhizome traits in 2015–2018.

QTL name	Marker interval^*^	Chr.	Mapping population				
Major-effect loci							
*qRED1.2*	C0120340-C0126288	1		B1	B2	B3	C1 and C2
*qRED3.1*	C033245-C036812	3	A	B1	B2		C1 and C2
*qRED3.3*	C0328430-C0330261	3	A	B1	B2	B3	C1 and C2
*qRED4.1*	C0422030-C0425168	4					C1 and C2
*qRED4.2*	C0425168-C0429250	4	A				C1 and C2
Minor-effect loci							
*qRED1.1*	C0114447-C0116133	1					C1 and C2
*qRED2.1*	C024508-C025764	2					C1 and C2
*qRED2.2*	C0220919-C0223081	2					C1 and C2
*qRED3.2*	C0311540-C0313246	3	A	B1	B2		
*qRED5*	C0523912-C0525155	5					C1 and C2
*qRED6.1*	C064060-C066039	6					C1 and C2
*qRED6.2*	C0620469-C0624727	6					C1 and C2
*qRED11*	C1110050-C1114284	11	A				

Chr, chromosome. Population A, B1, B2, and B3 were mapped with EPGM method. Population C1 and C2 were mapped with SGM method.

* The InDel maker name based on physical position in chromosome.

Compared with EPGM, SGM, only individuals representing the phenotypic extremes (the high and low phenotypic tails) from the entire mapping population being selected for genotyping, has been shown to have significant advantages in terms of cost savings, with negligible disadvantages in power of QTL detection (Sun et al., 2010). Using SGM of 169 plants with extreme RED from one F_2_ population with 5,340 plants, 11 loci controlling rhizome growth were detected in population C1. After replanted vegetatively, locations and the LOD score of QTLs in population C2 were general consistent with that in C1 (Figure 5D). Four loci *qRED1.2*, *qRED3.1*, *qRED3.3*, and *qRED4.2* were repeatedly detected by both EPGM and SGM methods and one locus *qRED4.1* with high LOD score was identified in population C1 and C2, while other loci were only detected by either EPGM or SGM (Figure 5). Considering the repeatability and LOD score of loci, five loci were regard as major-effect loci, while other loci were regard as minor-effect loci (Table 1; Supplementary Table S1). It was found that the scores of these five loci were higher than those of other loci by weighted algorithm (Supplementary Table S2). Compared with EPGM method, more minor-effect loci were identified by SGM method, and six of these loci could not be detected by EPGM (Table 1; Figure 5).

## Discussion

Environmental factors, such as light, temperature, soil, water, and mineral nutrients, greatly influence plant growth. Spatio-temporal heterogeneity of environmental factors is a pervasive feature in all natural habitats (Wiens, 1976; Smith and Vrieze, 1979; Turkington et al., 1991; Oborny and Kun, 2003; Turkington and Klein, 2011), and phenotypic plasticity of plants allows them to adjust their growth and development to cope with this challenge (de Kroon et al., 2005; Magyar et al., 2007). The behavior of plants to exploit favorable patches and avoid unfavorable patches *via* the selective placement of ramets has been interpreted as “foraging” response (Bell, 1984; Slade and Hutchings, 1987a,b; Sutherl and Stillman, 1988; Kelly, 1990, 1992; Piqueras et al., 1999). Ramets of some colonial plants can alter their step size (i.e., internode lengths of rhizome or stolon, also called spacer lengths) or branching intensity according to resource level (de Kroon and Hutchings, 1995). A major form of foraging is selective placement of ramets in resource-rich patches through decreased step size and increased branching intensity in resource-rich patches, while increased step size and decreased branching intensity are produced to escape resource-poor patches (Slade and Hutchings, 1987b). Shorter step size enable rapid habitat occupation and efficient resource use under strong interspecific competition pressure, while longer step size is useful to colonize new habitats when local resources are insufficient (Cain et al., 1996; Yun et al., 2014). Foraging behavior of clonal plants would confer an adaptive benefit since they can cope with spatio-temporal heterogeneity of environmental factors (de Kroon and Schieving, 1990; Hutchings and de Kroon, 1994). For better understanding of developmental mechanism of rhizome, it may be more feasible to study rhizome from an ecological perspective, not from an agronomic one. Branching intensity may increase in resource-rich patches, which means branching intensity is in accordance with resource level. So, branching intensity is a kind of “passive” growth response that would exist regardless of any fitness advantages. However, step size may decrease in resource-rich patches, which means step size is contrary to resource level. So step size response is an active process of habitat selection that would confer adaptive advantages to plants (Slade and Hutchings, 1987b; de Kroon and Schieving, 1990; Cain, 1994; de Kroon and Hutchings, 1995; Cain et al., 1996; Macek and Lepš, 2003). In summary, spacer (rhizome or stolon) length is generally more important for resource searching (i.e., foraging) than ramet number, which depends on branching intensity, so we pay more attention on RED. In fact, the number of tiller or rhizome is related to dormancy and germination of axillaries buds which depend mainly on apical dominance (Ali and Fletcher, 1970; Cline, 1991).

*Rhz2* and *Rhz3* were considered to be a pair of dominant complementary genes and largely control rhizomatousness in *O. longistaminata* (Hu et al., 2003). But further fine mapping results (Hu, 2010) implied that there should be more loci involved in rhizome growth. It has been hypothesized that there are multiple genes regulating the growth of rhizome. In this research, we employed EPGM and SGM methods to identify QTLs, regulating rhizome extension distance with three F_2_ populations in 4 years (Figure 1). Based on our results, there were 13 loci related to rhizome growth (Table 1; Figure 4), but not all of them were necessary simultaneously for rhizome growth. By comparing these two methods, it showed that results of SGM method are more stable and accurate; less affected by environment, and are better than EPGM to detect loci related to rhizome growth (Table 1). RED phenotype was not always consistent once F_2_ populations with continuous phenotypic distribution were replanted, which bring difficulty to confirm QTL mapping results. Clonal plants usually have higher phenotypic plasticity than non-clonal plants (Fazlioglu and Bonser, 2016), which may be partly explained this inconsistency. Minor-effect loci were more difficult than major-effect loci to be identified. Most recurring minor-effect loci were detected by SGM, and only *qRED3.2* obtained by EPGM method (Table 1). Population C1 and C2 contained two groups with extreme RED, one with well-developed rhizomes and another with poor-developed rhizomes; phenotypes of individual plants were less affected by environment, which largely ensured the repetitiveness of mapping results.

Five major-effect loci were identified through both EPGM and SGM methods in 4 years (Table 1). Except *qRED3.3* and *qRED4.2*, the other loci *qRED1.2*, *qRED3.1*, and *qRED4.1* were partially overlapped with previous ones (Hu et al., 2003), which brings out their significance in controlling rhizome development of perennial rice. *qRED1.2*, *qRED3.1*, and *qRED3.3* could be detected in almost all populations (Figure 5), which means that they are more stable than other loci. *qRED1.2*, *qRED3.1*, and *qRED4.1* were partially overlapped with *QRl1*, *Rhz2*, and *Rhz3* (Hu et al., 2003), respectively. Closely linked *qRED4.1*, and *qRED4.2* were both detected in the population C1 and C2, with *qRED4.2* more adjacent to *Rhz* (Maekawa et al., 1998).

Rice rhizome is a kind of complex trait controlled by multiple loci, and minimum numbers of loci required for rhizome growth are still unclear. More than 10 QTL that control rhizome-related traits were identified, among which one locus that control rhizome distance (distance traveled from crown by most distal rhizome) were identified in *Sorghum halepense* (Paterson et al., 1995). Five loci that control the presence of rhizomes and the EL-ST (elongated underground stems other than rhizomes) were detected in wild relatives of maize (Westerbergh and Doebley, 2004). Seven loci related to rhizomes presence, five loci for rhizome number, and six loci for rhizome distance were obtained in a recombinant inbred line from *Sorghum bicolor* and *Sorghum propinquum* (Kong et al., 2015). Comparison of genome-wide QTL scans in two wildrye suggested that rhizome growth needed at least four genes including one dominant, one additive gene, and two recessive genes in perennial wildrye (Yun et al., 2014). Phenotype and genotype analysis of some individuals showed that only five major-effect loci were not enough to ensure the occurrence of rhizome without the presence of some minor-effect loci. In all F_2_ populations, individuals with RED of 10–20 cm carried at least three major-effect loci, while those with RED over 20 cm carried at least four major-effect loci, demonstrating the significance of major-effect loci. Population C1 and C2 comprised 101 individuals with well-developed rhizome, most of which carried all five major-effect loci, only a few individuals with four (regardless of a few missing genotypes). Individual rice plants with well-developed rhizomes carried at least three major-effect loci and a certain number of minor-effect loci in our three F_2_ populations, which remind us to focus also on minor-effect loci. Both major-effect and minor-effect loci are involved in rhizome growth.

Major- and minor-effect loci worked together to control rhizome growth. The genetic effect of one locus depends on genotype at other loci, while no one could ensure rhizome presence alone. We take the interaction of *qRED3.1* with *qRED4.1*, *qRED5* and *qRED6.2* in population A, B1 and C1 as example to explain interaction of loci (Supplementary Image 2). Genetic effect of *qRED3.1* was similar to that of *qRED4.1* in population A and C1 (Supplementary Figures S7, S9). Phenotypic effects of *qRED3.1* and *qRED4.1* at the presence of AA (*O. longistaminata* homozygous) or AB (heterozygous) genotype were larger than that of BB (*O. sativa* homozygous genotype). When either one locus (*qRED3.1* or *qRED4.1*) was BB, the individuals had poor-developed rhizome (Supplementary Figures S7, S9).

Genetic effect of minor-effect loci *qRED5* and *qRED6.2* depended mainly on genotype at major-effect loci *qRED3.1* (*Rhz2*). RED phenotypes of *qRED5* or *qRED6.2* were poor when the genotype of *qRED3.1* was BB (*O. sativa* heterozygous genotype), compared with genotype AA (*O. longistaminata* heterozygous genotype) or AB (heterozygous) at *qRED3.1*. But the effects of *qRED3.1* were less affected (Supplementary Figures S10–S15) when genotype of *qRED5* or *qRED6.2* was BB, which indicated that effects of *qRED3.1* was larger than that of *qRED5* and *qRED6.2* and *qRED3.1* was less influenced by dysfunction of *qRED5* and *qRED6.2*. Genetic effect of *qRED5* or *qRED6.2* was larger when genotypes of *qRED3.1* was AA or AB, compared with BB, which indicated that major- and minor-effect loci could enhance genetic effect of each other. QTL *qRED4.1* had greater influence on *qRED3.1* than *qRED5* or *qRED6.2* in population A and C1, showed that there was a strong interaction between *qRED3.1* and *qRED4.1*.

## Conclusion

In this study, the QTL regulating rhizomes were detected in three F_2_ populations in 4 years by using both EPGM and SGM methods. Results showed that multiple genes regulated development of rhizomes, with over 10 loci related to rhizome growth. It has been found that both major- and minor-effect loci worked together to control rhizome growth. Some major-effect loci and minor-effect loci were function-overlapped and some complemented in regulating rhizome growth, while none could solely assure the presence of rhizome. At last, five major-effect loci were identified including *qRED1.2*, *qRED3.1*, *qRED3.3*, *qRED4.1*, and *qRED4.2*.

## Data Availability Statement

The original contributions presented in the study are included in the article/Supplementary Material, further inquiries can be directed to the corresponding authors.

## Author Contributions

JY designed the research. ZF, KW, and JR performed the research. ZC and L-ZT contributed to field management and data analysis. ZF, KW, YF, and JY analyzed the data and wrote the paper. All authors contributed to the article and approved the submitted version.

### Conflict of Interest

The authors declare that the research was conducted in the absence of any commercial or financial relationships that could be construed as a potential conflict of interest.

## Footnotes

^1^http://rice.plantbiology.msu.edu/annotation_pseudo_current.shtml

^2^https://brcwebportal.cos.ncsu.edu/qtlcart/WQTLCart.htm

## Supplementary Material

Supplementary Image 1 LOD score of different population calculated by R/QTL and Windows QTL Cartographer 2.5.

Supplementary Image 2 Interaction between *qRED3.1*, *qRED4.1*, *qRED5* and *qRED6.2* in different populations.

Supplementary Table 1 LOD and effect value of each QTL loci.

Supplementary Table 2 Arrange of loci with QTL score.
