# Supplementary material for: Interactions Among Multiple Quantitative Trait Loci Underlie Rhizome Development of Perennial Rice
Source: Front Plant Sci. 2020 Nov 12;11:591157. doi: 10.3389/fpls.2020.591157 (PMC7689344; doi:10.3389/fpls.2020.591157)
Supplement: Supplementary Image 1 — LOD score of different population calculated by R/QTL and Windows QTL Cartographer 2.5. [file Image_1.pdf]

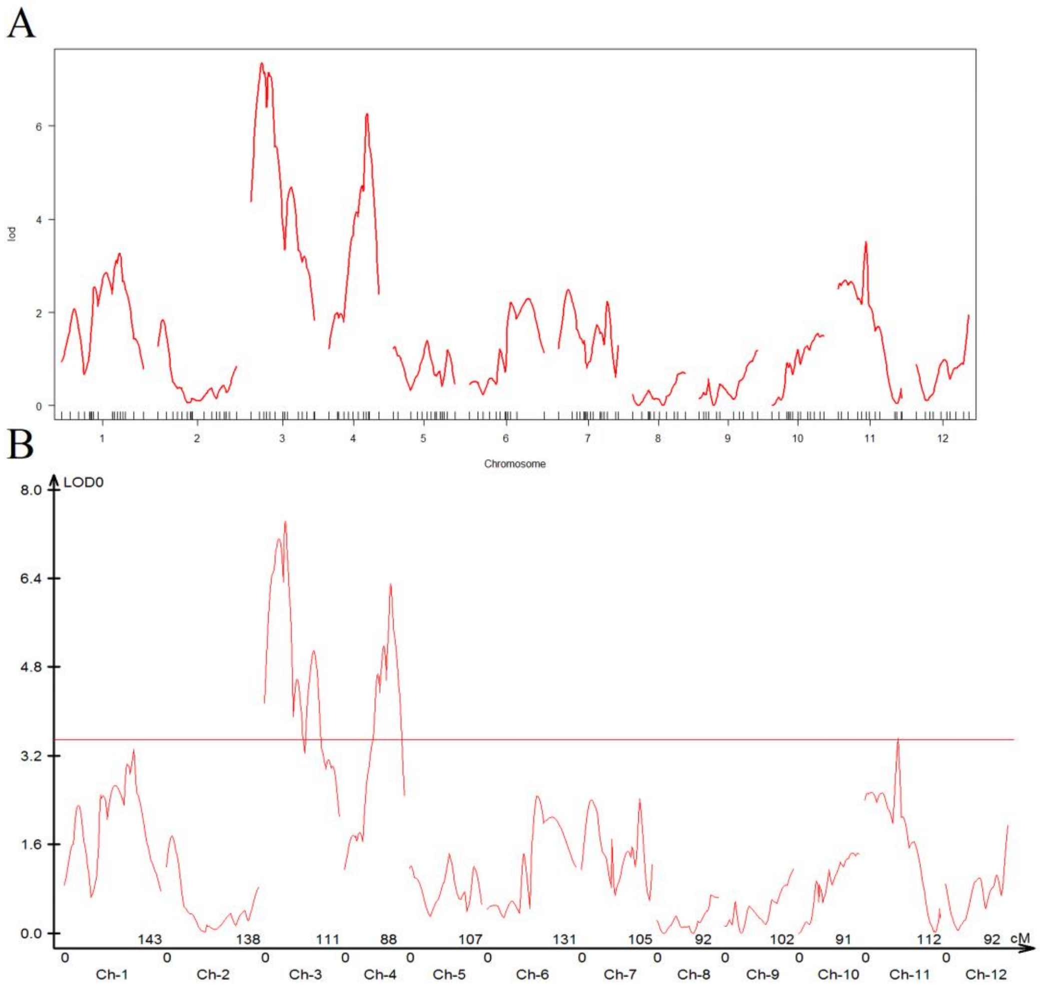

FIGURE S1 LOD score for QTL of population A. **(A)** calculated by R/QTL, **(B)** calculated by Windows QTL Cartographer 2.5.

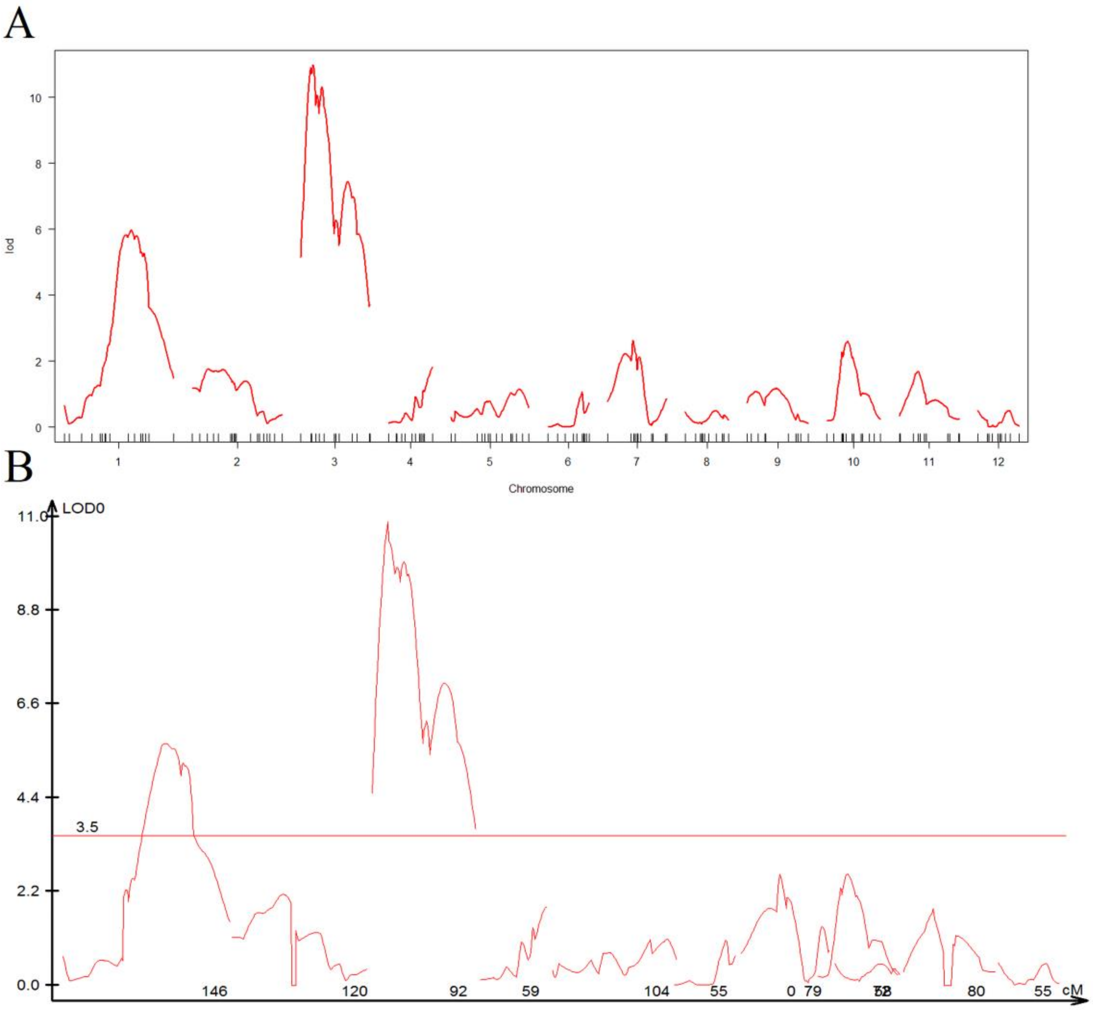

FIGURE S2 LOD score for QTL of population B1. **(A)** calculated by R/QTL, **(B)** calculated by Windows QTL Cartographer 2.5.

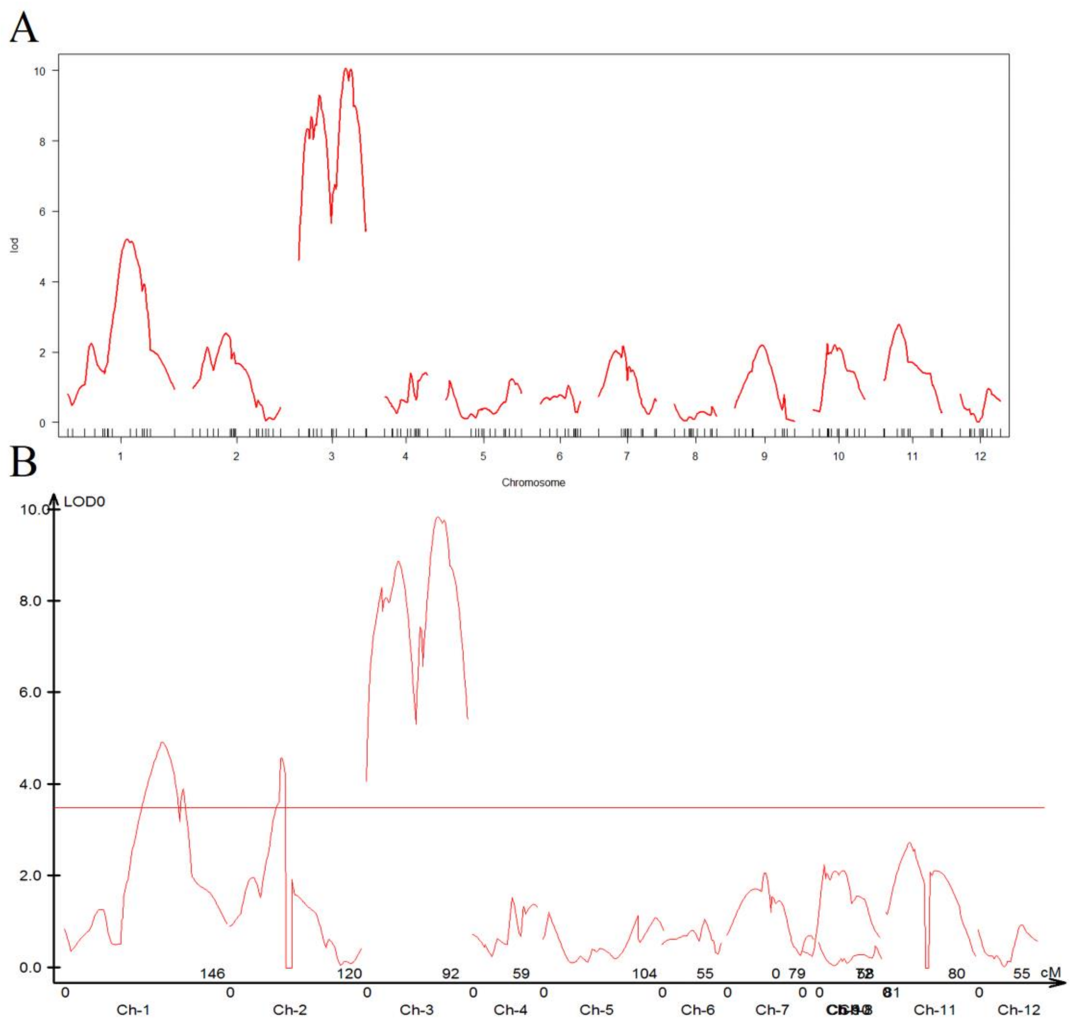

FIGURE S3 LOD score for QTL of population B2. **(A)** calculated by R/QTL, **(B)** calculated by Windows QTL Cartographer 2.5.

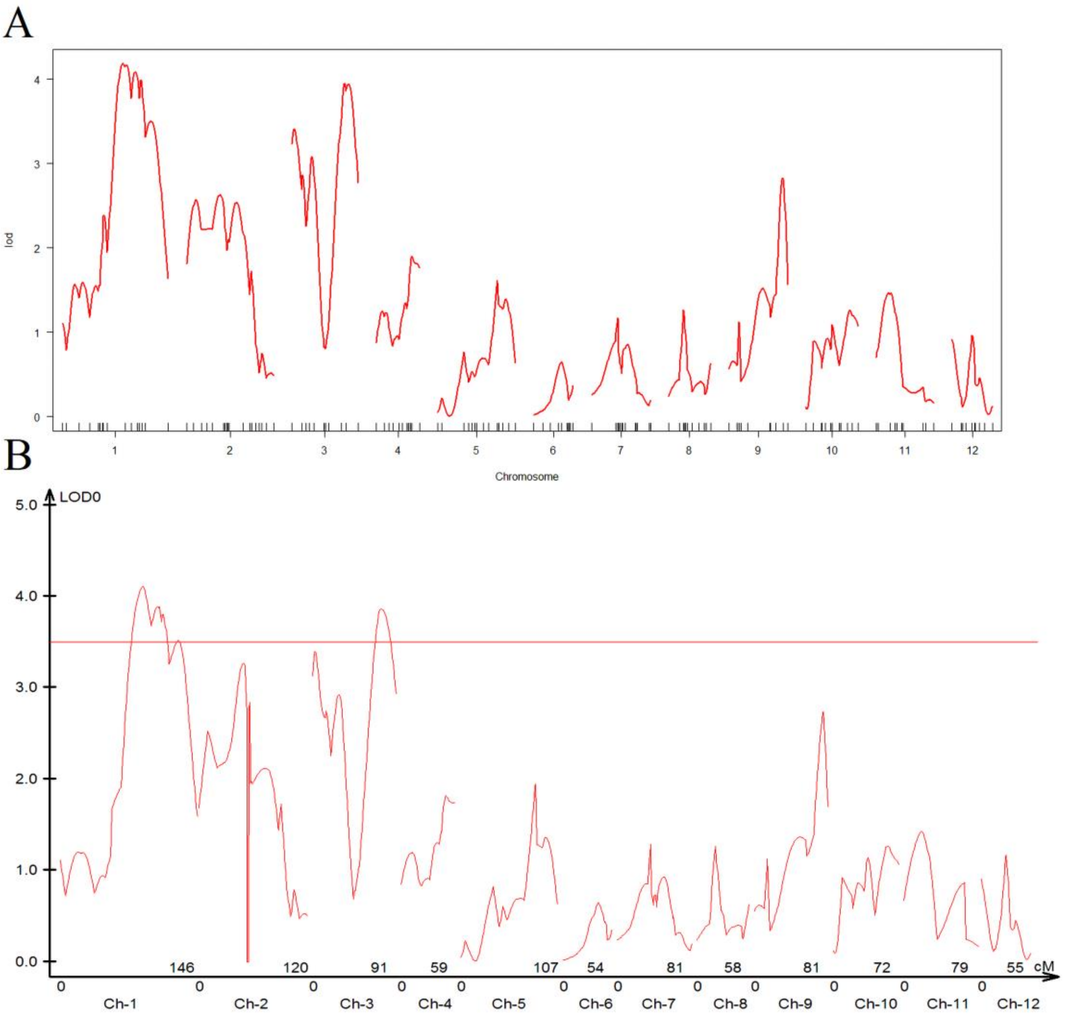

FIGURE S4 LOD score for QTL of population B3. **(A)** calculated by R/QTL, **(B)** calculated by Windows QTL Cartographer 2.5.

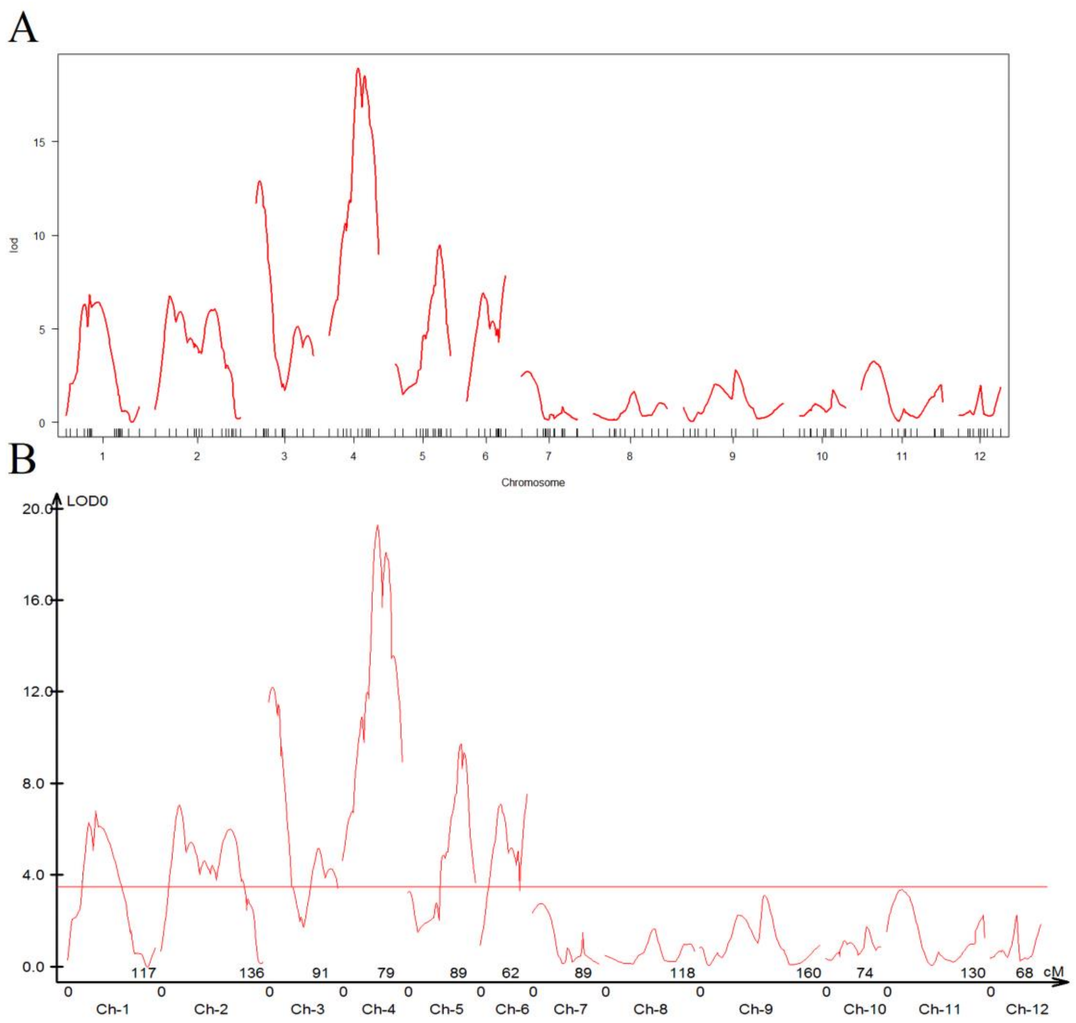

FIGURE S5 LOD score for QTL of population C1. **(A)** calculated by R/QTL, **(B)** calculated by Windows QTL Cartographer 2.5.

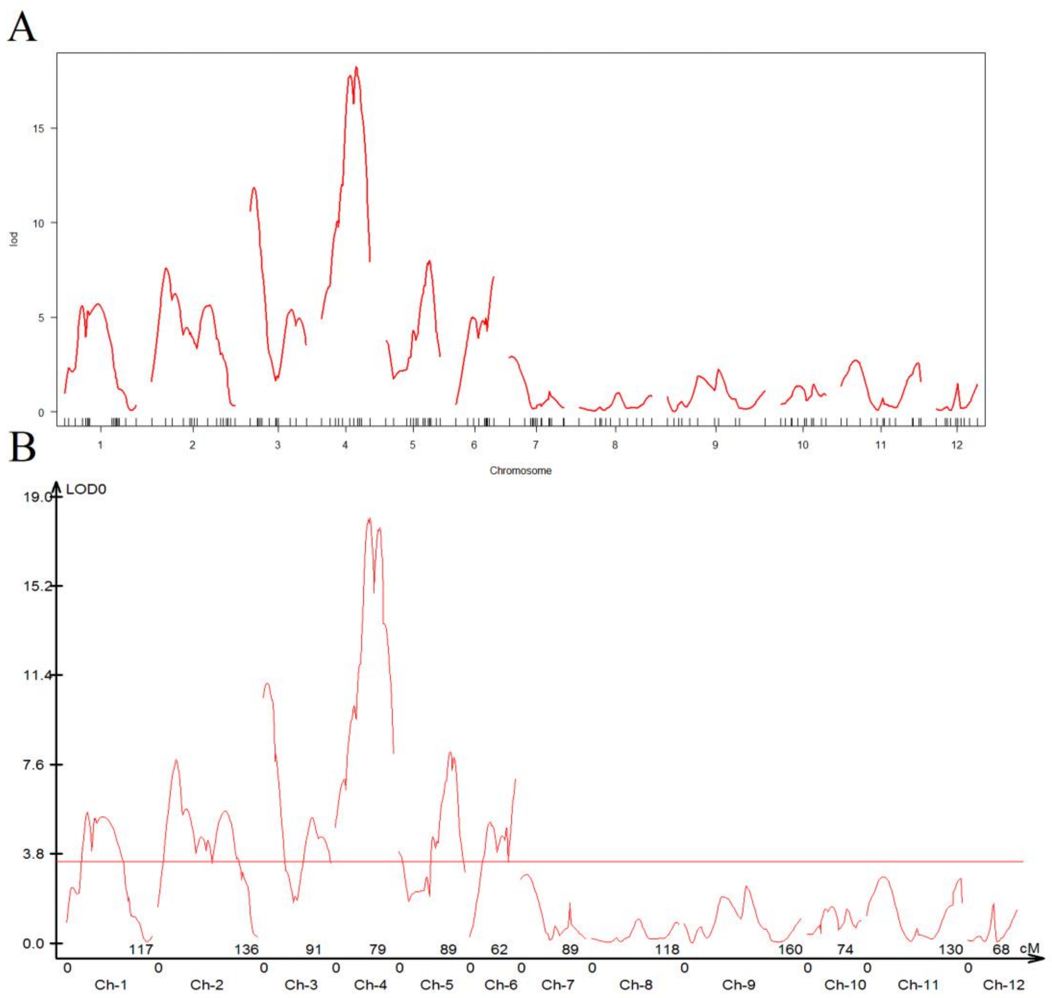

FIGURE S6 LOD score for QTL of population C2. **(A)** calculated by R/QTL, **(B)** calculated by Windows QTL Cartographer 2.5.
