# Supplementary material for: Interactions Among Multiple Quantitative Trait Loci Underlie Rhizome Development of Perennial Rice
Source: Front Plant Sci. 2020 Nov 12;11:591157. doi: 10.3389/fpls.2020.591157 (PMC7689344; doi:10.3389/fpls.2020.591157)
Supplement: Supplementary Image 2 — Interaction between qRED3.1, qRED4.1, qRED5 and qRED6.2 in different populations. [file Image_2.pdf]

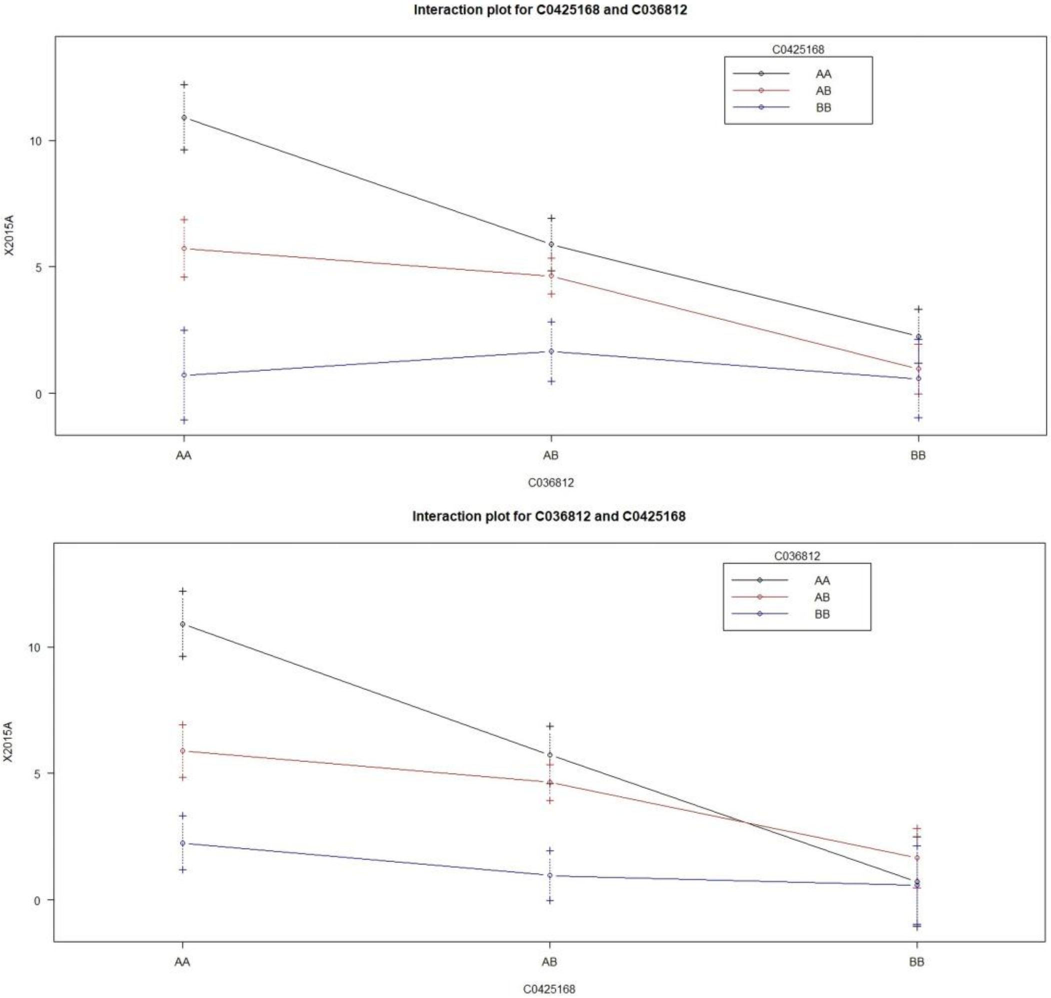

FIGURE S7 Interaction between *qRED3.1* and *qRED4.1* of population A. C036812: maker linked with *qRED3.1*, C0425168: maker linked with *qRED4.1*, genotype AA was *O. longistaminata* homozygous genotype, AB was heterozygous, BB was *O. sativa* homozygous genotype.

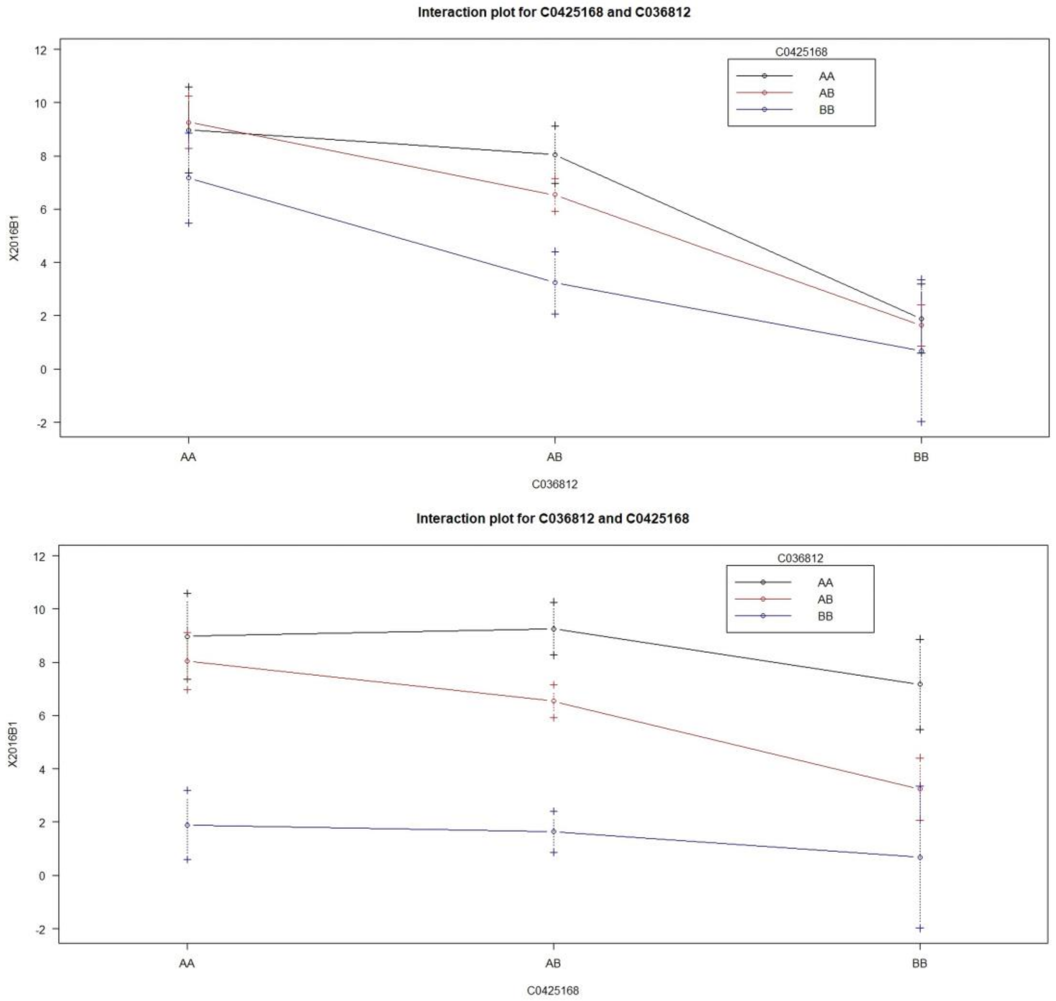

FIGURE S8 Interaction between *qRED3.1* and *qRED4.1* of population B1. C036812: maker linked with *qRED3.1*, C0425168: maker linked with *qRED4.1*, genotype AA was *O. longistaminata* homozygous genotype, AB was heterozygous, BB was *O. sativa* homozygous genotype.

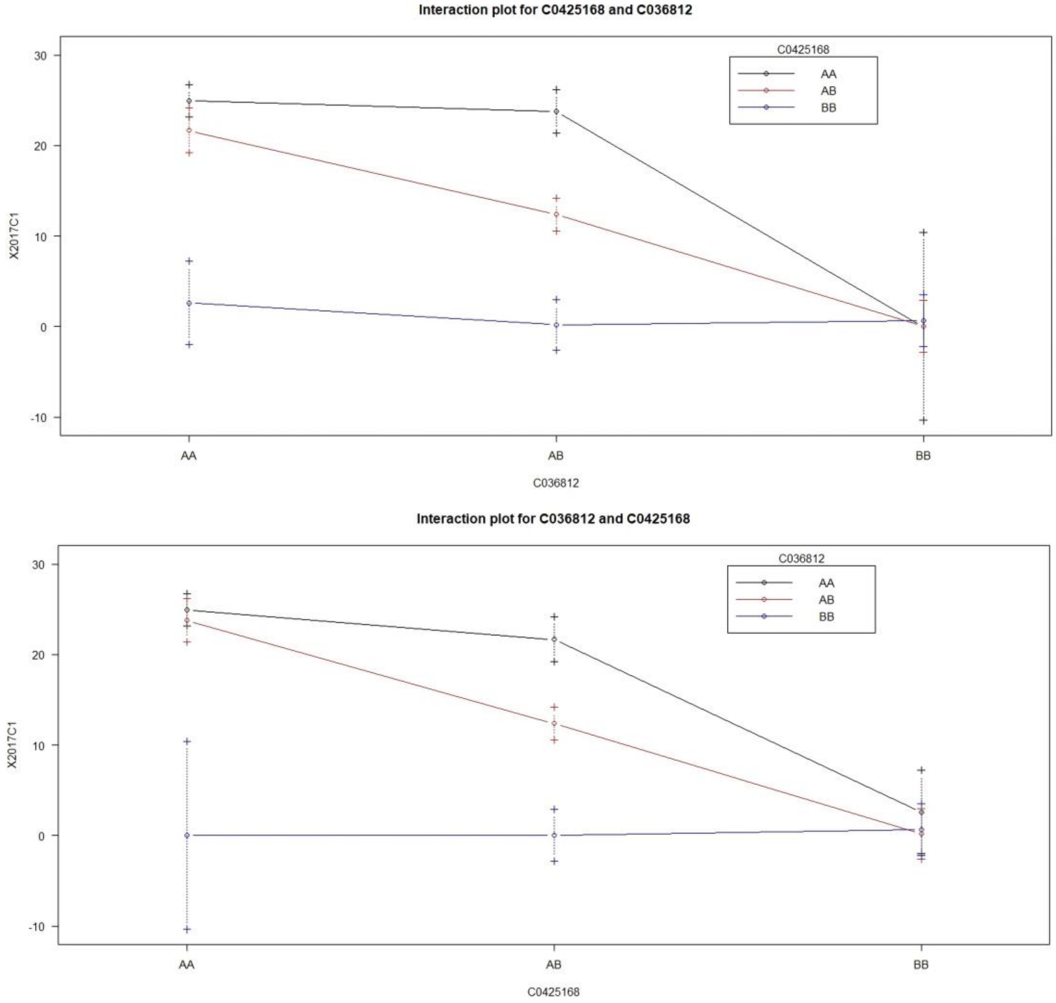

FIGURE S9 Interaction between *qRED3.1* and *qRED4.1* of population C1. C036812: maker linked with *qRED3.1*, C0425168: maker linked with *qRED4.1*, genotype AA was *O. longistaminata* homozygous genotype, AB was heterozygous, BB was *O. sativa* homozygous genotype.

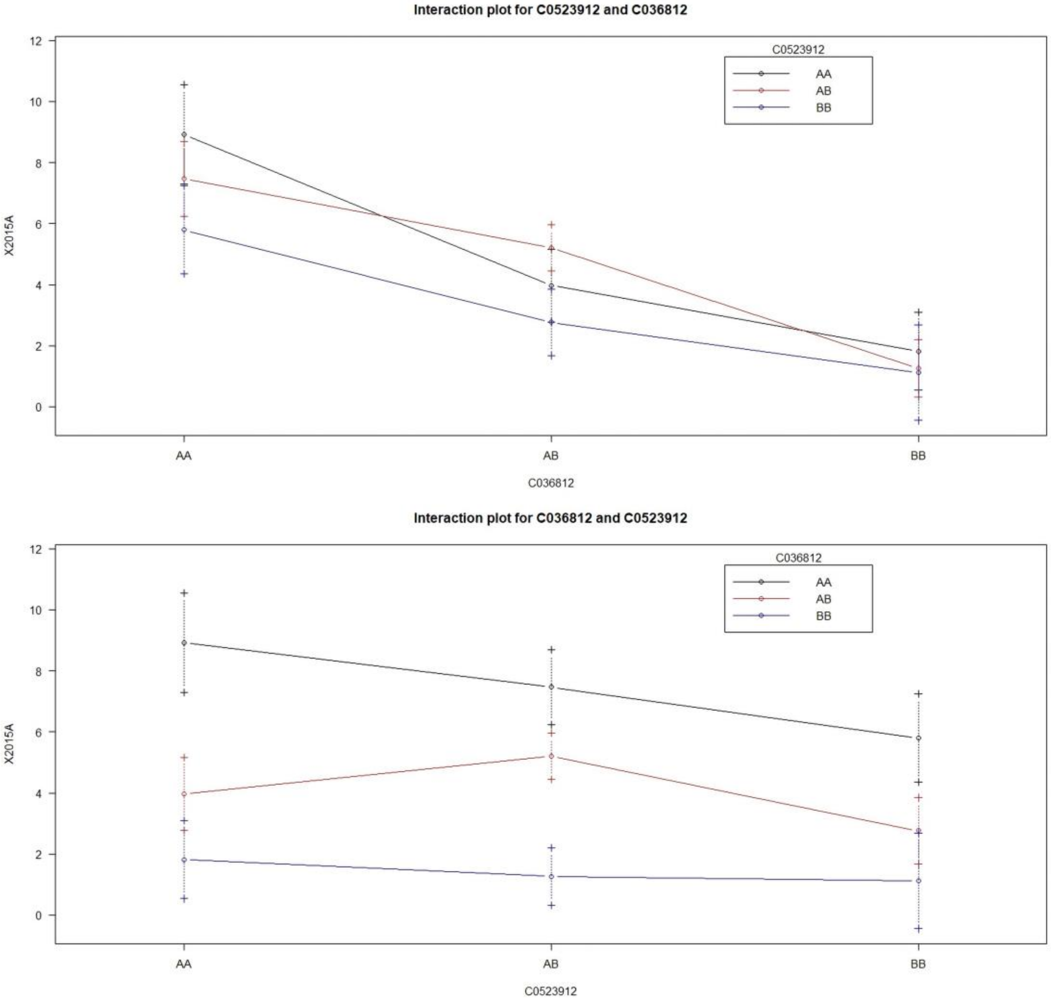

FIGURE S10 Interaction between *qRED3.1* and *qRED5* of population A. C036812: maker linked with *qRED3.1*, C0523912: maker linked with *qRED5*, genotype AA was *O. longistaminata* homozygous genotype, AB was heterozygous, BB was *O. sativa* homozygous genotype.

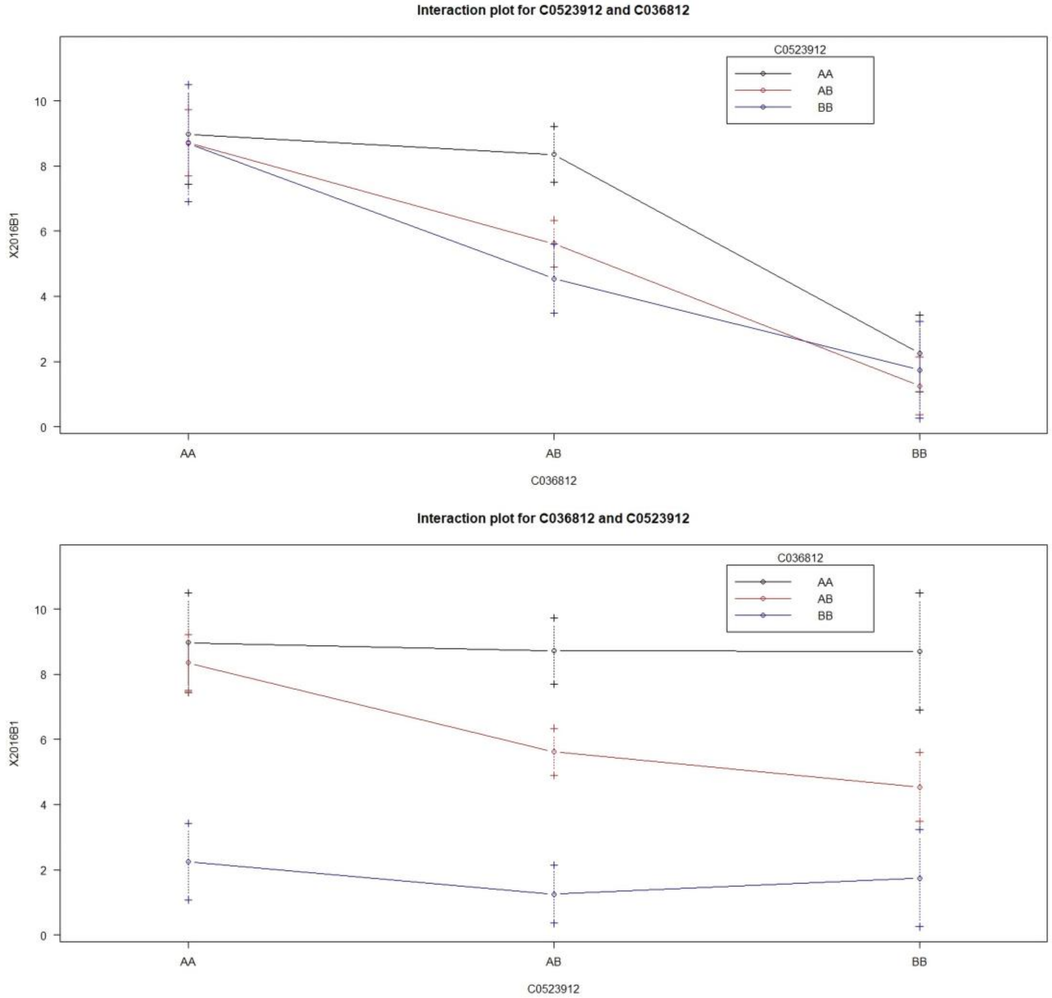

FIGURE S11 Interaction between *qRED3.1* and *qRED5* of population B1. C036812: maker linked with *qRED3.1*, C0523912: maker linked with *qRED5*, genotype AA was *O. longistaminata* homozygous genotype, AB was heterozygous, BB was *O. sativa* homozygous genotype.

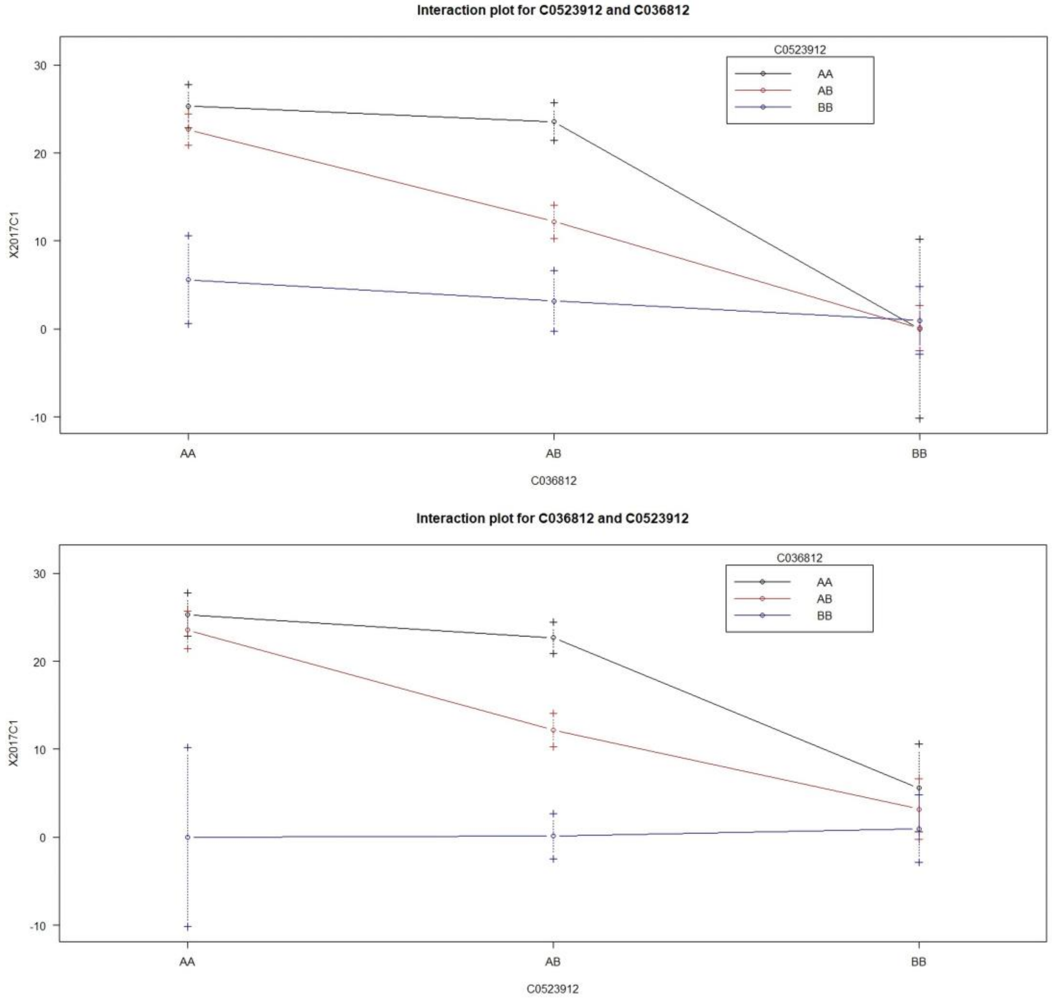

FIGURE S12 Interaction between *qRED3.1* and *qRED5* of population C1. C036812: maker linked with *qRED3.1*, C0523912: maker linked with *qRED5*, genotype AA was *O. longistaminata* homozygous genotype, AB was heterozygous, BB was *O. sativa* homozygous genotype.

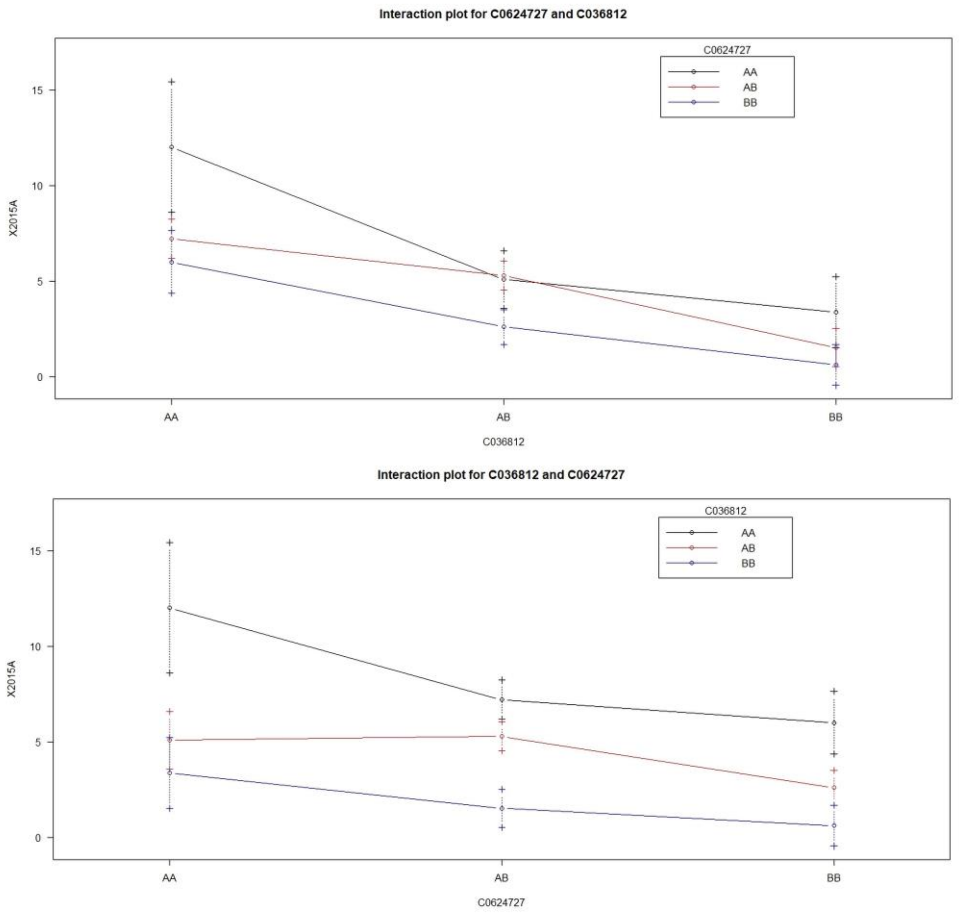

FIGURE S13 Interaction between *qRED3.1* and *qRED6.2* of population A. C036812: maker linked with *qRED3.1*, C0624727: maker linked with *qRED6.2*, genotype AA was *O. longistaminata* homozygous genotype, AB was heterozygous, BB was *O. sativa* homozygous genotype.

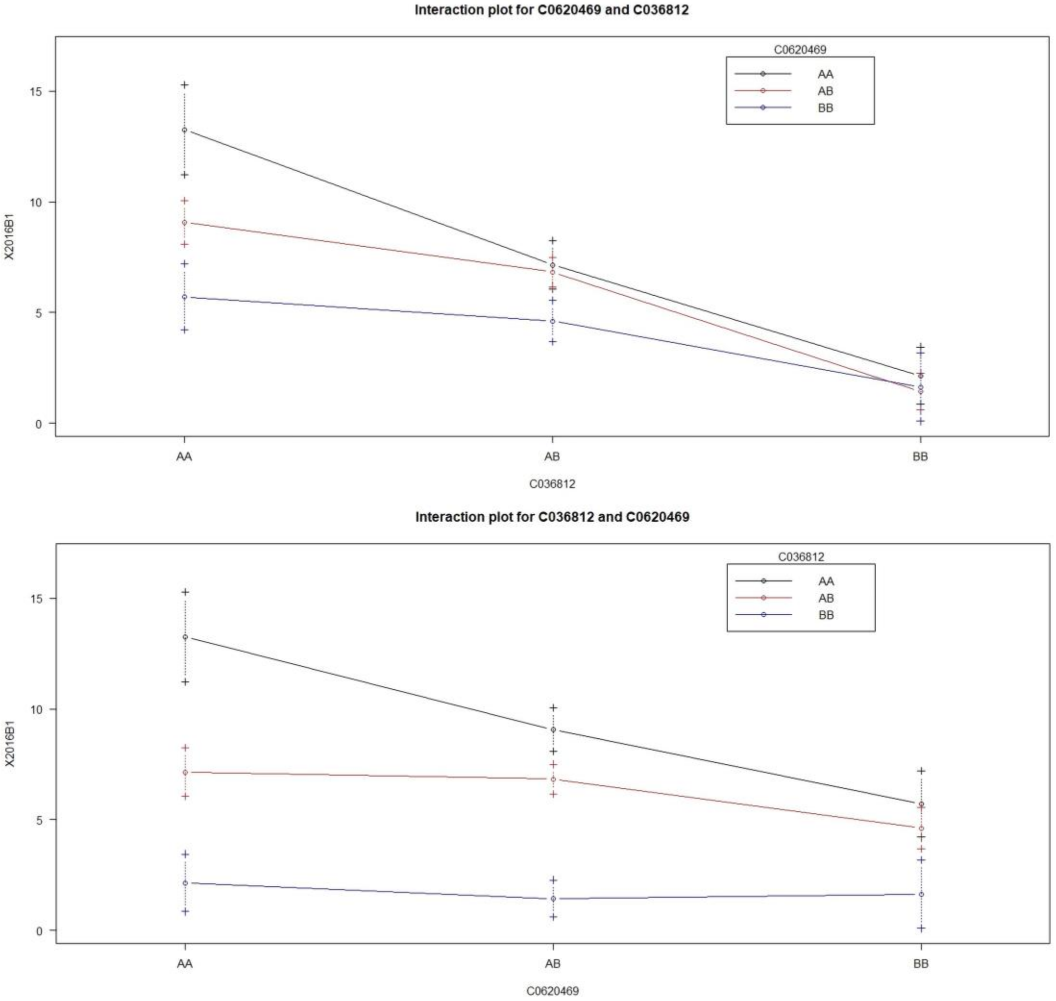

FIGURE S14 Interaction between *qRED3.1* and *qRED6.2* of population B1. C036812: maker linked with *qRED3.1*, C0620469: maker linked with *qRED6.2*, genotype AA was *O. longistaminata* homozygous genotype, AB was heterozygous, BB was *O. sativa* homozygous genotype.

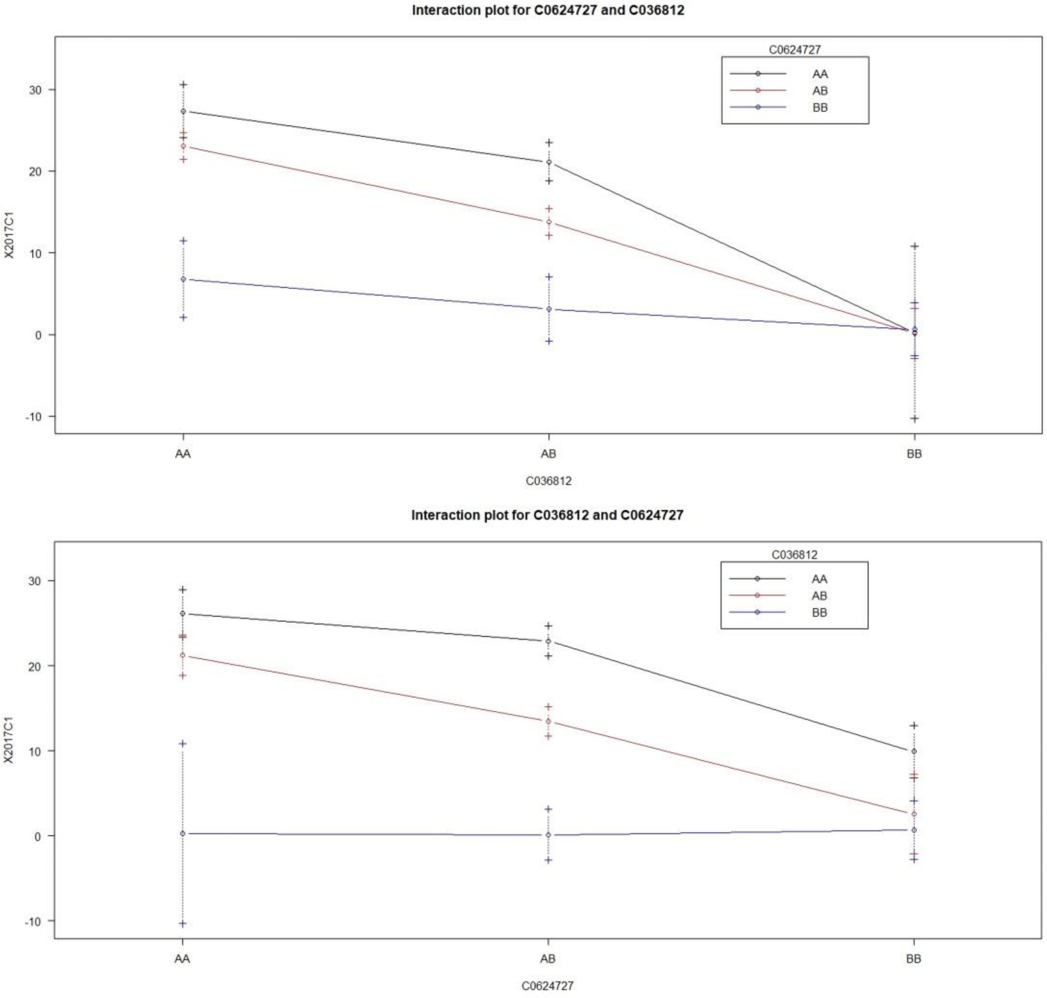

FIGURE S15 Interaction between *qRED3.1* and *qRED6.2* of population C1. C036812: maker linked with *qRED3.1*, C0624727: maker linked with *qRED6.2*, genotype AA was *O. longistaminata* homozygous genotype, AB was heterozygous, BB was *O. sativa* homozygous genotype.
