# Supplementary material for: Interactions Among Multiple Quantitative Trait Loci Underlie Rhizome Development of Perennial Rice
Source: Front Plant Sci. 2020 Nov 12;11:591157. doi: 10.3389/fpls.2020.591157 (PMC7689344; doi:10.3389/fpls.2020.591157)
Supplement: Supplementary Table 1 — LOD and effect value of each QTL loci. [file Table_1.doc]

**TABLE S1** The LOD and effect value of each QTL loci

| Mapping population | QTL name | LOD1 | LOD2 | Chromosome | Additive effect | Dominant effect | PVE |
| --- | --- | --- | --- | --- | --- | --- | --- |
| 2015 A | *qRED3.1* | 7.35 | 7.11 | 3 | 3.282 | 0.041 | 0.171 |
|  | *qRED3.2* | 7.15 | 7.41 | 3 | 3.585 | -0.542 | 0.180 |
|  | *qRED3.3* | 4.68 | 5.1 | 3 | 3.725 | -1.134 | 0.165 |
|  | *qRED4.2* | 6.26 | 6.29 | 4 | 3.437 | -1.404 | 0.152 |
|  | *qRED11* | 3.53 | 3.51 | 11 | 2.663 | -1.726 | 0.082 |
|  |  |  |  |  |  |  |  |
| 2016 B1 | *qRED1.2* | 5.96 | 5.65 | 1 | 1.473 | 1.945 | 0.102 |
|  | *qRED3.1* | 10.89 | 10.84 | 3 | 3.072 | 1.661 | 0.186 |
|  | *qRED3.2* | 10.06 | 9.94 | 3 | 3.374 | 1.064 | 0.179 |
|  | *qRED3.3* | 7.44 | 7.06 | 3 | 3.456 | -0.798 | 0.144 |
|  |  |  |  |  |  |  |  |
| 2016 B2 | *qRED1.2* | 5.21 | 4.9 | 1 | 2.237 | 1.733 | 0.093 |
|  | *qRED3.1* | 8.34 | 8.27 | 3 | 2.006 | 2.628 | 0.146 |
|  | *qRED3.2* | 9.3 | 8.87 | 3 | 3.338 | 1.038 | 0.160 |
|  | *qRED3.3* | 10.06 | 9.84 | 3 | 4.509 | -1.113 | 0.207 |
|  |  |  |  |  |  |  |  |
| 2017 B3 | *qRED1.2* | 4.18 | 4.09 | 1 | 1.492 | 1.514 | 0.081 |
|  | *qRED3.3* | 3.95 | 3.86 | 3 | 2.042 | 0.400 | 0.073 |
|  |  |  |  |  |  |  |  |
| 2017 C1 | *qRED1.1* | 6.82 | 6.78 | 1 | 7.385 | 3.444 | 0.169 |
|  | *qRED1.2* | 6.41 | 5.85 | 1 | 7.014 | 2.183 | 0.199 |
|  | *qRED2.1* | 6.75 | 7.05 | 2 | 10.791 | -8.609 | 0.208 |
|  | *qRED2.2* | 6.05 | 6.01 | 2 | 4.200 | 4.007 | 0.153 |
|  | *qRED3.1* | 12.9 | 12.2 | 3 | 9.738 | -2.747 | 0.305 |
|  | *qRED3.3* | 5.12 | 5.14 | 3 | 4.849 | 0.992 | 0.142 |
|  | *qRED4.1* | 18.7 | 19.26 | 4 | 11.913 | -1.811 | 0.448 |
|  | *qRED4.2* | 18.37 | 18.02 | 4 | 11.470 | -1.519 | 0.430 |
|  | *qRED5* | 9.48 | 9.73 | 5 | 10.295 | -2.911 | 0.253 |
|  | *qRED6.1* | 6.89 | 7.08 | 6 | 8.079 | 1.075 | 0.211 |
|  | *qRED6.2* | 7.82 | 7.56 | 6 | 8.260 | 0.474 | 0.212 |
|  |  |  |  |  |  |  |  |
| 2018 C2 | *qRED1.1* | 5.63 | 5.57 | 1 | 6.138 | 5.041 | 0.148 |
|  | *qRED1.2* | 5.7 | 5.4 | 1 | 6.617 | -0.479 | 0.204 |
|  | *qRED2.1* | 7.59 | 7.82 | 2 | 9.325 | -7.272 | 0.247 |
|  | *qRED2.2* | 5.66 | 5.65 | 2 | 3.366 | 3.105 | 0.145 |
|  | *qRED3.1* | 11.86 | 11.06 | 3 | 6.999 | -0.789 | 0.278 |
|  | *qRED3.3* | 5.4 | 5.33 | 3 | 4.230 | 0.559 | 0.143 |
|  | *qRED4.1* | 17.8 | 18.1 | 4 | 9.441 | -1.054 | 0.439 |
|  | *qRED4.2* | 18.24 | 17.67 | 4 | 8.782 | 0.021 | 0.413 |
|  | *qRED5* | 8 | 8.15 | 5 | 7.471 | -1.675 | 0.215 |
|  | *qRED6.1* | 5 | 5.17 | 6 | 5.131 | 1.532 | 0.159 |
|  | *qRED6.2* | 7.16 | 7.01 | 6 | 5.953 | 1.199 | 0.200 |

Note: LOD1 was calculated by R/qtl program, LOD2 and genetic effects were calculated by Windows QTL Cartographer 2.5.
