# Supplementary material for: Interactions Among Multiple Quantitative Trait Loci Underlie Rhizome Development of Perennial Rice
Source: Front Plant Sci. 2020 Nov 12;11:591157. doi: 10.3389/fpls.2020.591157 (PMC7689344; doi:10.3389/fpls.2020.591157)
Supplement: Supplementary Table 2 — Arrange of loci with QTL score. [file Table_2.doc]

Considering the repeatability and LOD of loci, the QTL identified in all populations were arranged by QTL score obtained by weighted algorithm (LOD and times of QTL detected account for 50% separately). The calculation formula is shown as the following.

QTL score= 0.5*
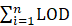
+ 0.5*n (n was the times of QTL detected).

**TABLE S2** The arrange of loci with QTL score.

| QTL name | LOD calculated by R/qtl | | | | | | Times of QTL | QTL score |
| --- | --- | --- | --- | --- | --- | --- | --- | --- |
|  | A | B1 | B2 | B3 | C1 | C2 |  |  |
| *qRED3.1* | 7.35 | 10.89 | 8.34 |  | 12.90 | 11.86 | 5 | 28.17 |
| *qRED4.2* | 6.26 |  |  |  | 18.37 | 18.24 | 3 | 22.935 |
| *qRED3.3* | 4.68 | 7.44 | 10.06 | 3.95 | 5.12 | 5.40 | 6 | 21.325 |
| *qRED4.1* |  |  |  |  | 19.26 | 18.10 | 2 | 19.68 |
| *qRED1.2* |  | 5.96 | 5.21 | 4.18 | 6.41 | 5.70 | 5 | 16.23 |
| *qRED3.2* | 7.15 | 10.06 | 9.30 |  |  |  | 3 | 14.755 |
| *qRED5* |  |  |  |  | 9.48 | 8.00 | 2 | 9.74 |
| *qRED6.2* |  |  |  |  | 7.82 | 7.16 | 2 | 8.49 |
| *qRED2.1* |  |  |  |  | 6.75 | 7.59 | 2 | 8.17 |
| *qRED1.1* |  |  |  |  | 6.82 | 5.63 | 2 | 7.225 |
| *qRED6.1* |  |  |  |  | 6.89 | 5.00 | 2 | 6.945 |
| *qRED2.2* |  |  |  |  | 6.05 | 5.66 | 2 | 6.855 |
| *qRED11* | 3.53 |  |  |  |  |  | 1 | 2.265 |
